# Rutaecarpine prevents hypertensive cardiac hypertrophy involving the inhibition of Nox4‐ROS‐ADAM17 pathway

**DOI:** 10.1111/jcmm.14308

**Published:** 2018-12-26

**Authors:** Si‐yu Zeng, Li Yang, Hui‐qin Lu, Qiu‐jiang Yan, Ling Gao, Xu‐ping Qin

**Affiliations:** ^1^ Department of Drug Clinical Trial Guangdong Second Provincial General Hospital Guangzhou China; ^2^ Laboratory of Vascular Biology, Institute of Pharmacy and Pharmacology University of South China Hengyang China; ^3^ Department of Cardiac & Thoracic Surgery The Third Affiliated Hospital of Guangzhou Medical University Guangzhou China; ^4^ Department of Pharmacy Guangdong Second Provincial General Hospital Guangzhou China

**Keywords:** a disintegrin and metalloproteinase‐17, extracellular signal‐regulated kinase ½, hypertensive cardiac hypertrophy, NADPH oxidase 4, rutaecarpine

## Abstract

Rutaecarpine attenuates hypertensive cardiac hypertrophy in the rats with abdominal artery constriction (AAC); however, its mechanism of action remains largely unknown. Our previous study indicated that NADPH oxidase 4 (Nox4) promotes angiotensin II (Ang II)‐induced cardiac hypertrophy through the pathway between reactive oxygen species (ROS) and a disintegrin and metalloproteinase‐17 (ADAM17) in primary cardiomyocytes. This research aimed to determine whether the Nox4‐ROS‐ADAM17 pathway is involved in the protective action of rutaecarpine against hypertensive cardiac hypertrophy. AAC‐induced hypertensive rats were adopted to evaluate the role of rutaecarpine in hypertensive cardiac hypertrophy. Western blotting and real‐time PCR were used to detect gene expression. Rutaecarpine inhibited hypertensive cardiac hypertrophy in AAC‐induced hypertensive rats. These findings were confirmed by the results of in vitro experiments that rutaecarpine significantly inhibited Ang II‐induced cardiac hypertrophy in primary cardiomyocytes. Likewise, rutaecarpine significantly suppressed the Nox4‐ROS‐ADAM17 pathway and over‐activation of extracellular signal‐regulated kinase (ERK) 1/2 pathway in the left ventricle of AAC‐induced hypertensive rats and primary cardiomyocytes stimulated with Ang II. The inhibition of Nox4‐ROS‐ADAM17 pathway and over‐activation of ERK1/2 might be associated with the beneficial role of rutaecarpine in hypertensive cardiac hypertrophy, thus providing additional evidence for preventing hypertensive cardiac hypertrophy with rutaecarpine.

## 1 INTRODUCTION

Hypertension, also named high blood pressure, causes enormous social and economic burden throughout the world. High blood pressure induces pathological cardiac hypertrophy, usually characterized by an enlarged cardiomyocyte, an increased protein synthesis and the reactivation of the foetal gene program. Nevertheless, ongoing high blood pressure promotes the transition from adaptive hypertrophy to maladaptive hypertrophy, eventually leading to sudden death, malignant arrhythmia and heart failure.^1^ Hence, pathological cardiac hypertrophy is an important adverse sign for the cardiovascular events, and inhibiting pathological cardiac hypertrophy may slow or prevent the progress of hypertension to heart failure.

Rutaecarpine (8,13‐dihydroindolo‐(2′,3′:3,4)pyrido(2,1‐b)quinazolin‐5(7H)‐one) is extracted from the dried fruit of *Evodia rutaecarpa* (Juss) Benth, also known as traditional Chinese herb Wu‐Zhu‐Yu used for antihypertensive therapy.^2^ Rutaecarpine was shown to cause vasorelaxing in a concentration‐dependent manner in rat aortic rings, inhibit anaphylaxis‐induced vasoconstriction in guinea‐pigs, and diminish blood pressure in anaesthetized rats.^3^, ^4^ Furthermore, rutaecarpine inhibits an increase in systolic blood pressure in phenol‐induced hypertensive rats and spontaneously hypertensive rats.^5^, ^6^ In addition, our previous studies have demonstrated that rutaecarpine reduces systolic blood pressure in renovascular hypertensive rats.^7^, ^8^ Taken together, these data suggest that rutaecarpine could be a potent antihypertensive agent.

Recently, rutaecarpine was shown to alleviate hypertensive cardiac hypertrophy in the rats subjected to abdominal artery constriction (AAC).^9^ High blood pressure contributes to the development of cardiac hypertrophy. A variety of studies have stated that rutaecarpine reduces blood pressure by increasing the synthesis and release of calcitonin gene‐related peptide (CGRP) and reducing the levels of angiotensin II (Ang II) as well as prolylcarboxypeptidase, a degrading enzyme of Ang II, in mesenteric artery of renohypertensive rats.^8^, ^10^ These findings suggest that the depressor effect plays a key role in the beneficial action of rutaecarpine against hypertensive cardiac hypertrophy. Besides its depressor effect, it still needs to further elucidate about how rutaecarpine inhibits hypertensive cardiac hypertrophy. Our previous research indicated that NADPH oxidase 4 (Nox4) promotes Ang II‐induced cardiac hypertrophy via the pathway of reactive oxygen species (ROS)‐a disintegrin and metalloproteinase‐17 (ADAM17) in cultured primary cardiomyocytes.^11^ Therefore, this study aimed to determine whether the Nox4‐ROS‐ADAM17 pathway is involved in the protective role of rutaecarpine in hypertensive cardiac hypertrophy in the rats subjected to AAC.

## 2 MATERIALS AND METHODS

### 2.1 Reagents

Rutaecarpine (84‐26‐4) was purchased from a commercial service (Chengdu Man‐Si‐Te Biological Technology Co., Ltd, Sichuan, China). Antibodies against the proteins were listed as follows: ADAM17 antibody (ab173579; Abcam, Shanghai, China), Nox4 antibody (14347‐1‐AP; Protentech, Wuhan, China), extracellular signal‐regulated kinase (ERK) 1/2 antibody (#4695; Cell Signaling Technology, Shanghai, China), phosphorylated ERK1/2 antibody (#4370; Cell Signaling Technology), GAPDH antibody (ab37168; Abcam), collagen I antibody (col I, GB11022‐1; Servicebio, Wuhan, China), collagen III antibody (col III, 13548‐1‐AP; Proteintech, Wuhan, China), tumour necrosis factor‐α antibody (TNF‐α, AF7014; Affinity, Changzhou, China), and Goat Anti‐Rabbit lgG HRP (S0001; Affinity). Foetal bovine serum (10099133) and Dulbecco's Modified Eagle Medium (DMEM, 12800017) were obtained from Thermo Fisher Scientific (Shanghai, China). Ang II (A9525) and TRITC‐labeled phalloidin (p1951) were provided by Sigma Aldrich (Shanghai, China).

### 2.2 Animals

All experimental procedures involving animals were performed according to the guidelines principles for the Care and Use of Laboratory Animals issued by the United States National Institutes of Health. The Animal protocols were simultaneously approved by the Medical Ethics Committee of Guangdong Second Provincial General Hospital. A total of 56 Sprague Dawley rats (male; weight, about 200 g) and 80 Sprague‐Dawley rats (1‐ to 3‐day‐old) were provided by the Experimental Animal Center of Sun Yat‐sen University.

### 2.3 Animal model of AAC‐induced cardiac hypertrophy

Abdominal artery constriction was carried out to induce pathological cardiac hypertrophy through pressure overload, as previously described.^12^, ^13^ In brief, the following procedures were described: rats were anaesthetized with sodium pentobarbital through intraperitoneal injection before the surgery until toe pinch reflex disappeared. Subsequently, a 5‐0 suture was tied twice around the suprarenal abdominal aorta where a 22‐gauge needle was inserted, and then the needle was removed to yield a 70%‐80% constriction of the abdominal aorta. Sham‐operated rats were created under the similar procedures without aorta banding. The rats were then randomly assigned to the following four groups: Sham‐operated rats treated with vehicle, AAC‐induced hypertensive rats treated with vehicle, AAC‐induced hypertensive rats treated with low dose of rutaecarpine (Rut (L), 20 mg/kg/day), AAC‐induced hypertensive rats treated with high dose of rutaecarpine (Rut (H), 40 mg/kg/day), with 10 rats in each group. The dosage of rutaecarpine used is based on our previous studies.^7^, ^8^

### 2.4 Measurement of blood pressure

Systolic blood pressure (SBP) was measured in conscious state before the treatment with rutaecarpine, at the 2nd week and the 4th week after the treatment using tail‐cuff method.^14^

### 2.5 Echocardiographic evaluation

Transthoracic echocardiography was conducted using a Vevo 2100 High‐Resolution In Vivo Microimaging System (Visual Sonics, Toronto, ON, Canada) as previously described.^12^, ^13^, ^15^ After the rats were anaesthetized in ultrasonic atomization with 2% isoflurane, all good‐quality images were obtained to measure left ventricular internal diameter (LVID) during diastole or systole, left ventricular anterior wall thickness (LVAW) during diastole or systole, left ventricular posterior wall thickness (LVPW) during diastole or systole, fractional shortening (FS) and ejection fraction (EF).

### 2.6 Haemodynamic measurement

Haemodynamic measurement was implemented in the rats as previously described.^12^ After the rats were anaesthetized with sodium pentobarbital (ip, 45 mg/kg), a 24‐gauge polyethylene catheter filled with heparin was introduced into the right carotid artery of rats, and systolic arterial blood pressure (SABP), diastolic arterial blood pressure (DABP) were measured using a BL‐420S system (Chengdu Tai‐meng Technology Co., Ltd, Sichuan, China). Next, the 24‐gauge polyethylene catheter was further introduced into the left ventricle of rats. Finally, the BL‐420S system was used to measure the maximal rate of left ventricular pressure increase (dp/dtmax) and decrease (dp/dtmin), and heart rate.

### 2.7 Histological analysis

The hearts were arrested in diastole using potassium chloride (30 mmol/L), fixed with 10% formalin overnight, and then embedded in paraffin. Subsequently, transverse, transmural slices of the left ventricle (about 5 μm thickness) were prepared and placed on adhesive slides. Next, the slides were stained with haematoxylin and eosin (H&E) as well as Masson's trichrome reagent. Then, myocyte cross‐sectional area was measured in 10 randomly chosen nonrepeating fields in cross‐sections stained with H&E using Image‐pro plus 6.0 according to the method described previously.^16^ Finally, interstitial fibrosis was quantified as the percentage of fibrotic area over the total myocardial area in five randomly chosen nonrepeating visual fields (excluding the fields that contained a coronary artery) of sections stained with Masson's trichrome reagent using Image‐pro plus 6.0.

### 2.8 Immunohistochemistry

Immunohistochemistry was used to detect the protein levels of col I, col III and TNF‐α in the left ventricle as described previously.^17^

### 2.9 RNA isolation and quantitative real‐time quantitative PCR

RNA extraction and quantitative real‐time PCR were performed as described previously.^18^ Real‐time PCR used primers for atrial natriuretic peptide (ANP), brain natriuretic peptide (BNP), Col I, Col III, Nox4, ADAM17, TNF‐α and the gene‐specific for GADPH was chose as an inner control. The premiers used in this study are described as follows: ANP: 5'‐GGAAGTCAA CCCGTCTCA‐3' (forward primer) and 5'‐AGCCCTCAGTTTGCTTTT‐3' (reverse primer); BNP: 5'‐ATGCAGAAGCTGCTGGAGCTGATA‐3' (forward primer) and 5'‐TTGTAGGGCCTTG GTCCTTTGAGA‐3' (reverse primer); Col I: 5'‐GCCTCAAGGTATTGCTGGAC‐3' (forward premier) and 5'‐ACCTTGTTTGCCAGGTTCAC‐3' (reverse premier); Col III: 5'‐CTGGACCC CAGGGTCTTC‐3' (forward premier) and 5'‐CATCTGATCCAGGGTTTCCA‐3' (reverse premier); Nox4: 5'‐AGCTGCCCACTTGGTGAACGC‐3' (forward primer), 5'‐TCAGGCCCGGA ACAGTT GTGA‐3' (reverse primer); ADAM17: 5'‐GTGAGCAGTTTCTCGAACGC‐3' (forward primer) and 5'‐AGCTTCTCAAGTCGCAGGTG‐3' (reverse primer); TNF‐α: 5'‐TGGCGTGTTCATCCGTTC TC‐3' (forward primer), 5'‐CCCAGAGCCACAATTCCCTT‐3' (reverse primer); GADPH: 5'‐AT CAAGAAGGTGGTGAAGCA‐3' (forward primer), 5'‐AAGGTGGAAGAATGGGAGTTG‐3' (reverse primer).

### 2.10 Western blotting

Western blotting was executed according to the standard procedures previously described.^11^, ^19^

### 2.11 Cell culture and drug treatment

Primary ventricular cardiomyocytes were isolated from 1‐ to 3‐day‐old Sprague‐Dawley rats as described before.^20^ Ventricular cardiomyocytes were cultured in DMEM supplemented with 10% foetal bovine serum for 48 hours. To induce hypertrophy, cardiomyocytes were maintained in serum‐free DMEM for 24 hours and treated with 100 nmol/L Ang II for 24 hours. For determining the effect of rutaecarpine (10 μmol/L) on pathological cardiac hypertrophy, primary cardiomyocytes were treated with rutaecarpine for 60 minutes prior to the stimulation with Ang II. The concentration of rutaecarpine was chosen according to previous references.^21^, ^22^

### 2.12 Cell surface area

After fixed with 4% paraform, primary cardiomyocytes were permeabilized using 0.5% Triton X‐100 and then incubated with TRITC‐labeled phalloidin for 30 minutes at room temperature. Next, the cell surface area was automatically analysed in 40 nonrepeated fields of the cardiomyocytes using a Cellomics/High Content Screening (Thermo Scientific, Shanghai, China) as previously described.^11^, ^13^

### 2.13 Detection of hydrogen peroxide (H_2_O_2_) and superoxide (O^2−^)

Hydrogen peroxide (H_2_O_2_) level was detected using a hydrogen peroxide assay kit (S0038; Beyotime Biotechnology, Shanghai, China). The procedures were briefly described as follows: First, a standard curve was constructed using different H_2_O_2_ solutions and the responding values of optical density; Second, the samples obtained from the left ventricle or primary cardiomyocytes were prepared using cell lysis buffer and then used to detect the responding values of optical density under 520 nm; Third, the H_2_O_2_ level was calculated using the standard curve and the value of optical density.

The content of intracellular O^2−^ was determined using dihydroethidium (DHE; S0063; Beyotime Biotechnology). Confluent cardiomyocytes grown in 24‐well plate were pretreated with rutaecarpine (10 μmol/L) for 60 minutes before treating with Ang II. Cardiomyocytes were then incubated with 500 μL DMEM containing 5 μmol/L DHE for 30 minutes when avoiding light. After being washed with DMEM, the cells were used to detect DHE fluorescence intensity using a Cellomics/High Content Screening (Thermo Scientific).

### 2.14 Enzyme‐linked immunosorbent assay

After cardiomyocytes were stimulated with Ang II for 24 hours, the conditioned DMEM was collected to detect the mature TNF‐α released from myocardial cells. These samples were used to detect the TNF‐α level by a TNF‐α enzyme‐linked immunosorbent assay kit (CSB‐E11987r; Cusabio Biotech., Co. Ltd., Wuhan, China) according to the manufacturer's instructions.

### 2.15 Statistical analysis

Data are expressed as mean ± SD. Statistical analysis was performed using one‐way or two‐way ANOVA followed by Bonferroni's post hoc test among at least three groups, and *P < *0.05 was considered to have statistical significance.

## 3 RESULTS

### 3.1 Effect of rutaecarpine on blood pressure in the rats subjected to AAC

For evaluating the effect of rutaecarpine on blood pressure, we compared SBP, SABP, and DABP obtained from the four groups. As shown in Table 1, no significant difference was observed in SBP among the four groups before the operation. However, there were significant increases in SBP (116 ± 7.4 mm Hg vs 180 ± 10.6 mm Hg), SABP (126 ± 7.6 mm Hg vs 184 ± 8.7 mm Hg), and DABP (79 ± 5.6 mm Hg vs 105 ± 7.4 mm Hg) in the AAC rats compared with those of the Sham group (Tables 1 and 2). These findings indicate the successful construction of a rat model of AAC‐induced hypertension. By contrast, treatment with low or high dosage of rutaecarpine for 4 weeks caused marked reductions in SBP, SABP and DABP in AAC‐induced hypertensive rats (Tables 1 and 2). Likewise, the AAC‐induced hypertensive rats treated with low or high dosage of rutaecarpine exhibited increases in dp/dtmax and dp/dtmin, but no significant change in heart rate compared with the AAC group. Accordingly, rutaecarpine decreased blood pressure in AAC‐induced hypertensive rats, consistently with its depressor effect reported by previous studies.^5^, ^6^, ^7^, ^8^, ^9^

**Table 1:** Effect of rutaecarpine (Rut) on systolic blood pressure (SBP, mm Hg) in the rats subjected to abdominal artery constriction (AAC)

SBP	0 week	2nd week	4th week
Sham	114 ± 6.8	118 ± 6.7	120 ± 8.8
AAC	116 ± 7.4	150 ± 7.8^#^	180 ± 10.6^#^
AAC + Rut (L)	118 ± 6.5	128 ± 8.4*	134 ± 9.6*
AAC + Rut (H)	116 ± 6.6	124 ± 7.5*	127 ± 7.2*

Data are mean ± SD.

The statistical tests were performed using two‐way ANOVA followed by post hoc test. n = 10.

^#^ *P* < 0.05 vs Sham group.

* *P* < 0.05 vs AAC group.

**Table 2:** Effect of rutaecarpine (Rut) on echocardiographic and haemodynamic parameters in the rats subjected to abdominal artery constriction (AAC)

	Sham	AAC	AAC + Rut (L)	AAC + Rut (H)
Electrocardiographic data				
LVAWd (mm)	1.63 ± 0.09	2.20 ± 0.15^#^	1.74 ± 0.07*	1.67 ± 0.10*
LVAWs (mm)	2.53 ± 0.19	3.53 ± 0.40^#^	2.87 ± 0.24*	2.61 ± 0.21*
LVPWd (mm)	1.67 ± 0.12	2.37 ± 0.25^#^	2.01 ± 0.09*	1.87 ± 0.09*
LVPWs (mm)	2.65 ± 0.13	3.80 ± 0.35^#^	3.03 ± 0.20*	2.83 ± 0.14*
LVIDd (mm)	7.38 ± 0.24	7.87 ± 0.35	7.43 ± 0.27	7.39 ± 0.31
LVIDs (mm)	4.47 ± 0.35	4.86 ± 0.26	4.63 ± 0.19	4.54 ± 0.32
Fractional shortening (%)	34.60 ± 3.29	39.30 ± 4.12	38.6 ± 4.93	37.9 ± 3.48
Ejection fraction (%)	63.46 ± 6.75	64.82 ± 13.11	66.4 ± 8.93	65.7 ± 7.38
Haemodynamic data				
SABP (mm Hg)	126 ± 7.6	184 ± 8.7^#^	138 ± 8.5*	132 ± 7.9*
DABP (mm Hg)	79 ± 5.6	105 ± 7.4^#^	89 ± 6.9*	84 ± 6.5*
Heart rate (beats/min)	355.0 ± 21.4	383.5 ± 27.2	353.5 ± 26.5	365.9 ± 29.2
dp/dtmax (mm Hg/s)	4.93 ± 0.21	3.47 ± 0.25^#^	4.36 ± 0.13*	4.47 ± 0.15*
dp/dtmin (mm Hg/s)	‐4.77 ± 0.31	‐3.30 ± 0.17^#^	‐4.33 ± 0.20*	‐4.56 ± 0.28*

LVAWd, left ventricular anterior wall thickness during diastole; LVAWs, left ventricular anterior wall thickness during systole; LVPWd, left ventricular posterior wall thickness during diastole; LVPWs, left ventricular posterior wall thickness during systole; LVIDd, LV internal diameter during diastole; LVIDs, LV internal diameter during systole; SABP, Systolic artery blood pressure; DABP, Diastolic artery blood pressure; dp/dtmax, the maximal rate of left ventricular pressure increase; dp/dtmin, the maximal rate of left ventricular pressure decrease.

Data are mean ± SD.

One‐way ANOVA followed by post hoc test was carried out for the statistical tests. n = 10.

^#^ *P* < 0.05 vs Sham group.

* *P* < 0.05 vs AAC group.

### 3.2 Rutaecarpine suppressed hypertensive cardiac hypertrophy

**Figure 1:**
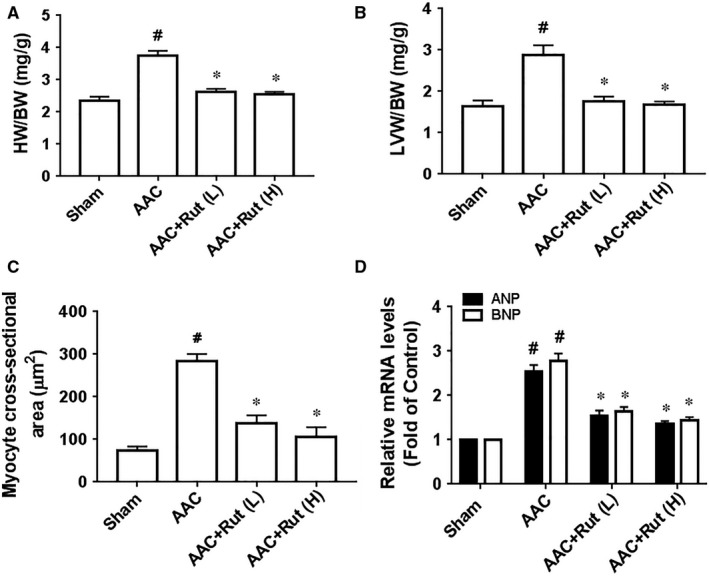
Rutaecarpine (Rut) inhibited hypertensive cardiac hypertrophy in the rats subjected to abdominal artery constriction (AAC). A, HW/BW (n = 9‐10 per group). B, LVW/BW (n = 9‐10 per group). C, Cross‐sections of the hearts from the rats subjected to AAC or sham operation were stained with haematoxylin & eosin to analyse myocyte cross‐sectional area (n = 5 per group). D, Real‐time PCR analyses of hypertrophic markers (ANP and BNP, n = 5 in each group). Rut (L) represents low dosage of rutaecarpine; Rut (H) represents high dosage of rutaecarpine; HW/BW represents the ratio between heart weight and body weight; LVW/BW represents the ratio between left ventricular weight and body weight. ^#^ *P* < 0.05 vs Sham group, *P < 0.05 vs SHR group. One‐way ANOVA followed by Bonferroni's post hoc test was carried out for the statistical tests

**Figure 2:**
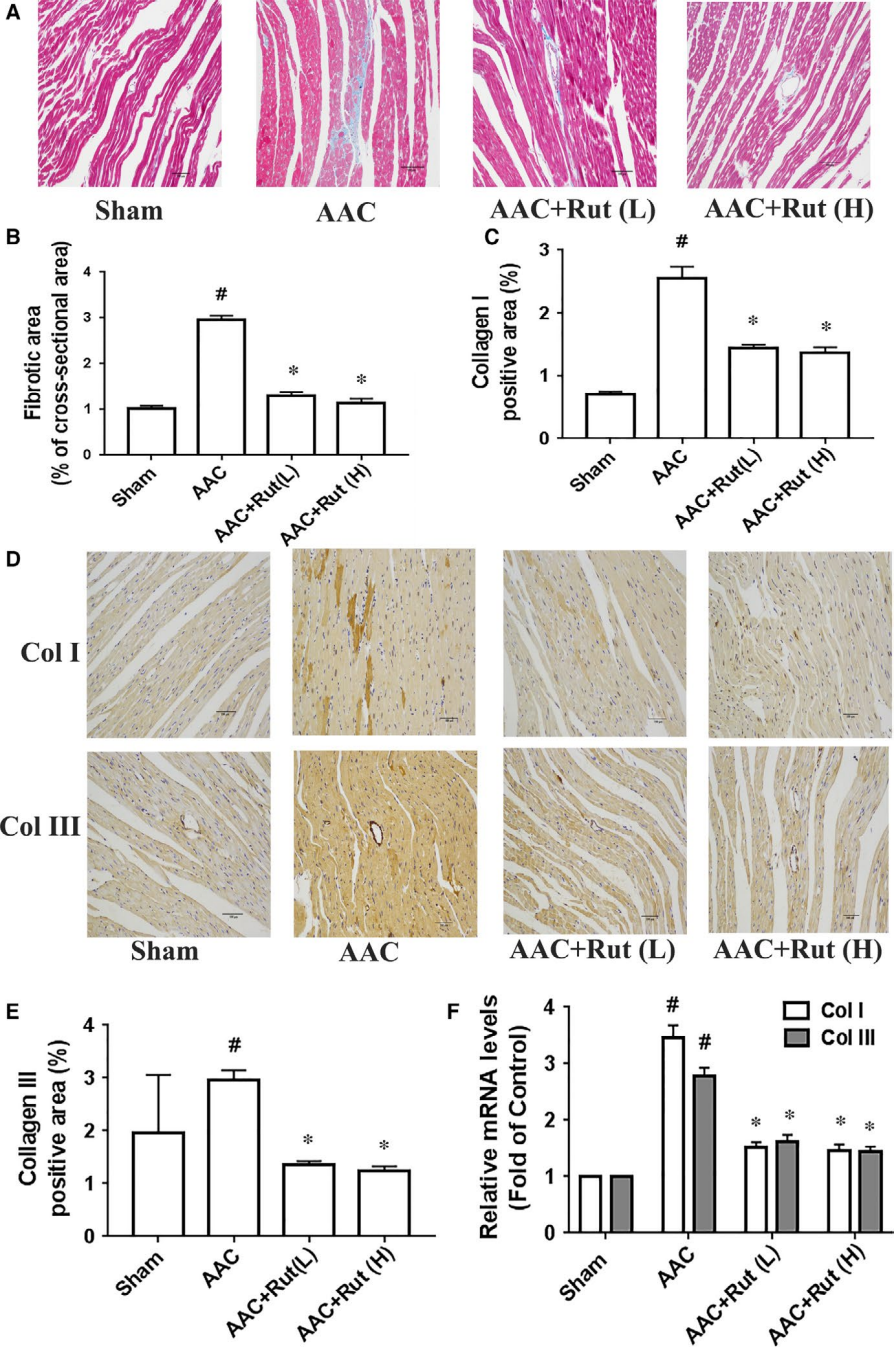
Rutaecarpine inhibited cardiac fibrosis in the rats subjected to abdominal artery constriction (AAC). A, Representative microphotographs of Masson staining of heart. B, Fibrosis area (n = 5 per group). C, Col I positive area (n = 5 per group). D, Representative microphotographs of immunochemistry for col I and col III. E, Col III positive area (n = 5 per group). F, mRNA levels of col I and col III in the left ventricle (n = 4 per group). One‐way ANOVA followed by Bonferroni's post hoc test was used for the statistical tests. Rut (L) represents low dosage of rutaecarpine; Rut (H) represents high dosage of rutaecarpine; Col I represents collagen I; col III represents collagen III. ^#^ *P* < 0.05 vs Control group; *P < 0.05 vs AAC group

We next investigated the role of rutaecarpine in hypertensive cardiac hypertrophy in AAC‐induced hypertensive rats. After 4 weeks of AAC induction, the ratio between heart weight and body weight (HW/BW), and the ratio between left ventricular weight and body weight (LVW/BW) were markedly elevated in the AAC rats compared with those of the Sham group (Figure 1A and B). Histological examination revealed an increase in myocyte cross‐sectional area in the AAC group compared with that of the Sham group (Figure 1C). Furthermore, the results of echocardiographic and haemodynamic analyses showed that the heart of AAC rat exhibited significant elevations in left ventricular anterior wall thickness during diastole (LVAWd) and systole (LVAWs), left ventricular posterior wall thickness during diastole (LVPWd) and systole (LVPWs) compared with the Sham group (Table 2). Concurrently, the mRNA levels of hypertrophic genes, such as ANP and BNP, were significantly higher in the AAC group than those of the Sham group (Figure 1D). These evidences suggest that hypertensive cardiac hypertrophy is induced through AAC induction for 4 weeks. In contrast to the results observed in the AAC rats, treatment with low or high dosages of rutaecarpine remarkably attenuated hypertensive cardiac hypertrophy, as indicated by a decrease in the HW/BW, the LVW/BW, the myocyte cross‐sectional area, the LVAWd, the LVAWs, the LVPWd, the LVPWs and the mRNA levels of hypertrophic genes, compared with those of the AAC group (Figure 1 and Table 2). Moreover, low or high dosage of rutaecarpine significantly decreased the fibrotic area and the protein and mRNA levels of col I and col III in the left ventricle of AAC‐induced hypertensive rats (Figure 2). Taken together, rutaecarpine alleviated hypertensive cardiac hypertrophy.

### 3.3 Rutaecarpine prevented Ang II‐induced cardiac hypertrophy in primary cardiomyocytes

**Figure 3:**
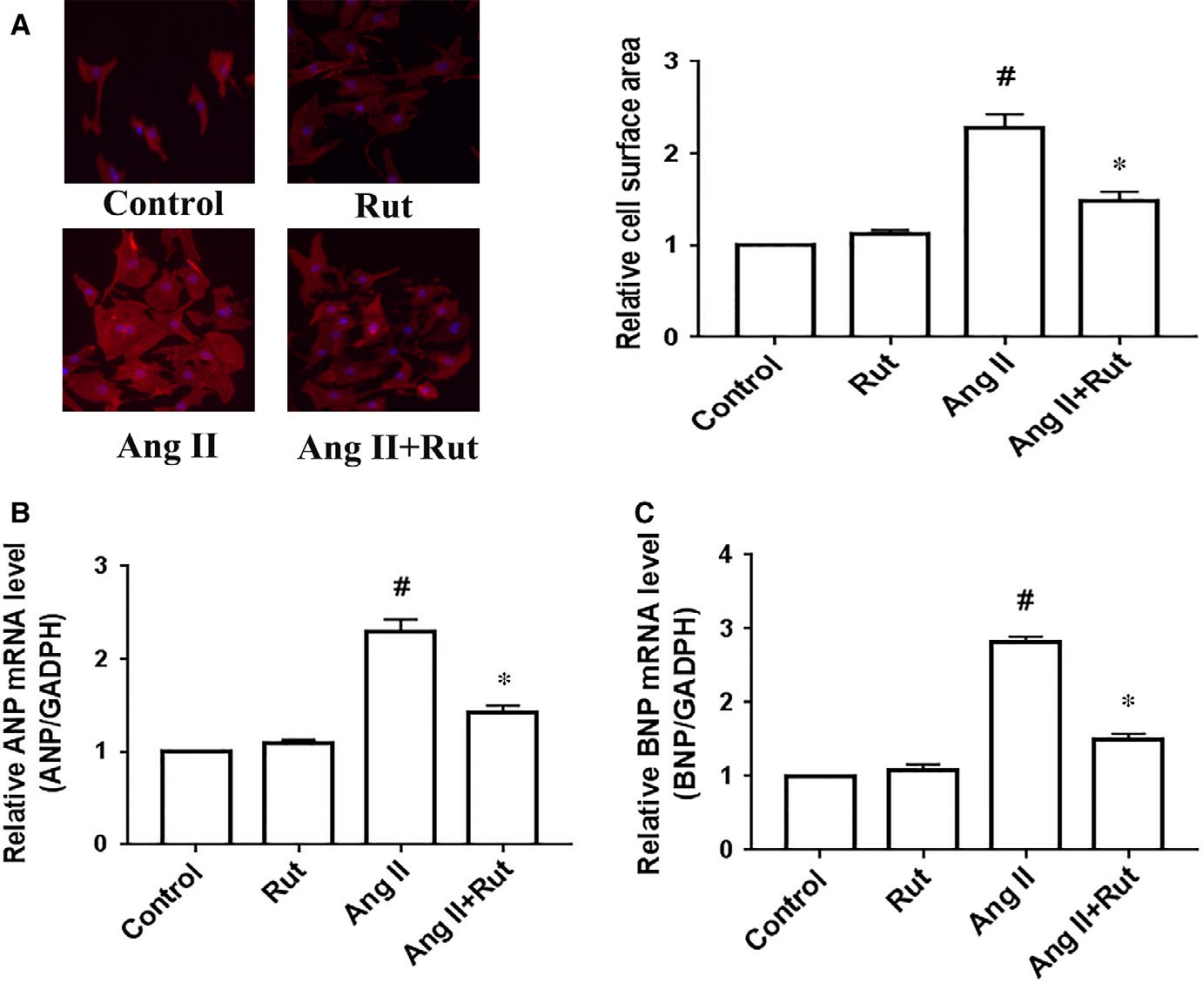
Rutaecarpine (Rut) alleviated angiotensin II (Ang II)‐induced cardiac hypertrophy in primary cardiomyocytes. Primary cardiomyocytes were treated with 100 nmol/L angiotensin II for 24 h after pretreating with rutaecarpine (10 µmol/L) for 60 min. A, Cell surface area. B and C, The mRNA levels of hypertrophic genes ANP (B) and BNP (C). ^#^ *P* < 0.05 vs control group; *P < 0.05 vs Ang II group. The statistical tests were undertaken by one‐way ANOVA followed by Bonferroni's post hoc test. n = 3 independent experiments

We subsequently observed the effect of rutaecarpine on pathological cardiac hypertrophy in vitro. In cardiomyocytes, pathological cardiac hypertrophy could be modeled through Ang II stimulation.^11^, ^16^, ^23^ Exposure to Ang II (100 nmol/L) increased cell surface area and the expression of established hypertrophic genes, for example, ANP and BNP, whereas pretreatment with rutaecarpine (10 μmol/L) for 60 minutes significantly inhibited Ang II‐induced up‐regulation of the cell surface area and the mRNA levels of ANP and BNP in primary cardiomyocytes (Figure 3). These observations indicate that rutaecarpine reduces Ang II‐induced cardiac hypertrophy.

### 3.4 Rutaecarpine inhibited the Nox4‐ROS‐ADAM17 pathway in hypertrophic cardiomyocytes

**Figure 4:**
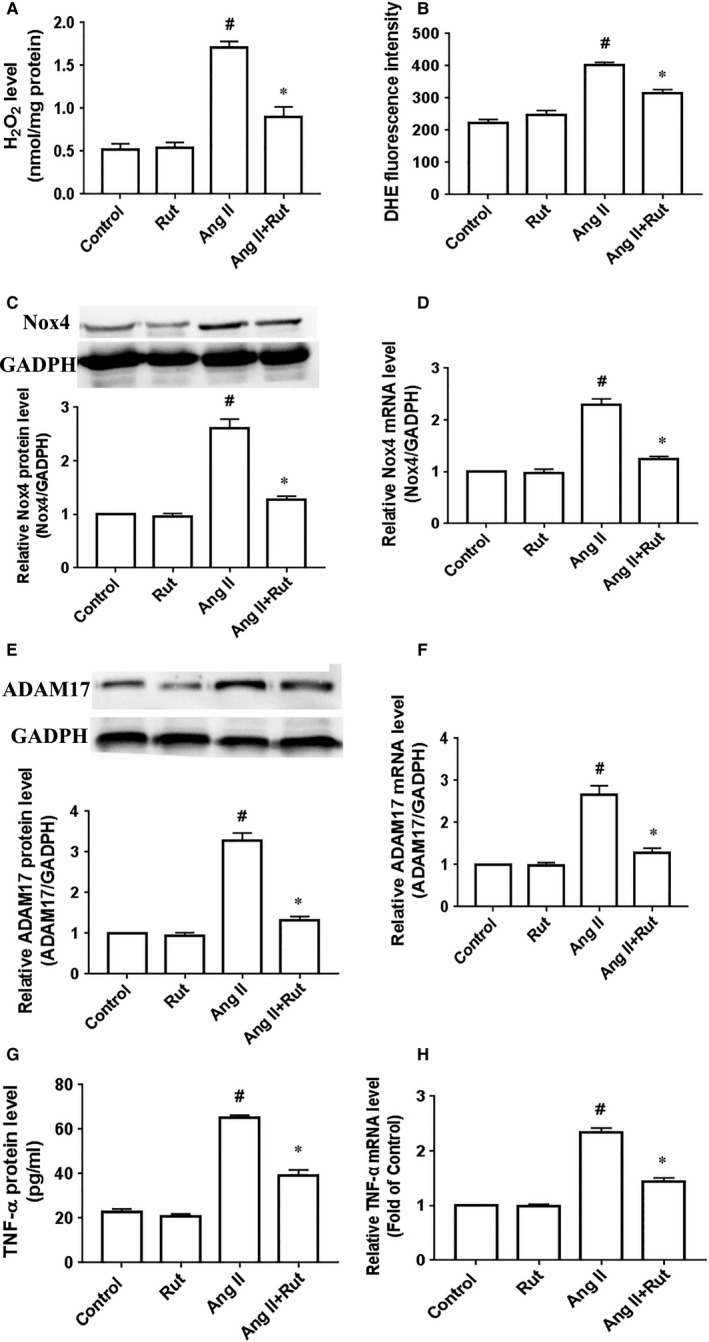
Rutaecarpine (Rut) inhibited the Nox4‐ROS‐ADAM17 pathway in cardiomyocytes treated by angiotensin (Ang II). Primary cardiomyocytes were treated with rutaecarpine (10 µmol/L) for 60 min prior to stimulation with Ang II for 24 h. A and B, The contents of H_2_O_2_ (A) and O^2−^ level indicated by DHE fluorescence intensity (B). C, Nox4 protein level. D, Nox4 mRNA level. E, ADAM17 protein level. F, ADAM17 mRNA level. G, TNF‐α protein level in the conditioned media. H, TNF‐α mRNA level. Nox4 represents NADPH oxidase 4; ROS represents reactive oxygen species; ADAM17 represents a disintegrin and metalloproteinase‐17; TNF‐α represents tumour necrosis factor α; ^#^ *P* < 0.05 vs Control group; *P < 0.05 vs Ang II group. The statistical analyses were performed using one‐way ANOVA followed by Bonferroni's post hoc test. n = 3 independent experiments

To further delineate mechanisms underlying the inhibitory effect of rutaecarpine against hypertensive cardiac hypertrophy, we determined whether rutaecarpine suppressed the Nox4‐ROS‐ADAM17 pathway in hypertrophic cardiomyocytes. Firstly, we examined the hypothesis in primary cardiomyocytes. As can be seen from Figure 4A and B, rutaecarpine down‐regulated the ROS production, indicated by the contents of O^2−^ and H_2_O_2_, in primary cardiomyocytes stimulated with 100 nmol/L Ang II for 24 hours. We next determined the effect of rutaecarpine on Nox4 expression that is a major resource for ROS in the heart. The Nox4 protein and mRNA levels were significantly elevated in primary cardiomyocytes treated with Ang II compared with those of the Control group, while rutaecarpine inhibited Ang II‐induced up‐regulation of Nox4 expression in cardiomyocytes (Figure 4C and D). Likewise, rutaecarpine markedly reduced ADAM17 expression in primary cardiomyocytes treated with Ang II (Figure 4D and F). Considering that ADAM17 is the sheddase that promotes the release of TNF‐α from the membrane via directly targeting pro‐TNF‐α,^24^ the TNF‐α protein level is usually used to evaluate the ADAM17 activity. As shown in Figure 4G, rutaecarpine significantly decreased the TNF‐α protein level in the conditioned media, suggesting the inhibitory effect of rutaecarpine on the ADAM17 activity. In addition, rutaecarpine also reduced TNF‐α mRNA level in cardiomyocytes treated with Ang II (Figure 4H). Our previous research demonstrated that Nox4 promoted cardiac hypertrophy via activating the ROS‐ADAM17 pathway in primary cardiomyocytes stimulated with 100 nmol/L Ang II for 24 hours.^11^ Therefore, rutaecarpine inhibits the Nox4‐ROS‐ADAM17 pathway in primary cardiomyocytes treated with Ang II.

**Figure 5:**
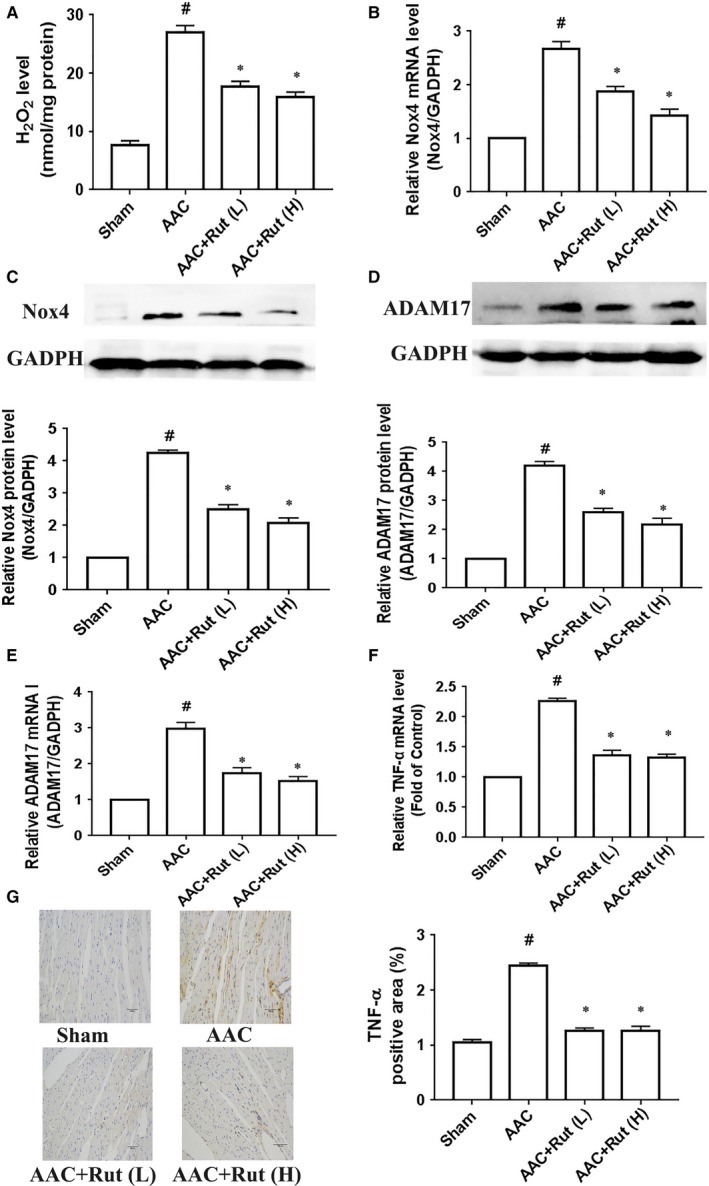
Rutaecarpine (Rut) suppressed the Nox4‐ROS‐ADAM17 pathway in the left ventricle of rats subjected to abdominal artery constriction (AAC). A, The H_2_O_2_ level (n = 5 per group). B, Nox4 mRNA level (n = 5 per group). C, Nox4 protein level (n = 4 per group). D, ADAM17 protein level (n = 4 per group). E, ADAM17 mRNA level; (n = 5 per group). F, TNF‐α mRNA level (n = 4 per group). G, TNF‐α protein level (n = 5 per group). Nox4 represents NADPH oxidase 4; ROS represents reactive oxygen species; ADAM17 represents a disintegrin and metalloproteinase‐17; TNF‐α represents tumour necrosis factor α; ^#^ *P* < 0.05 vs Sham group, *P < 0.05 vs SHR group. The statistical tests were carried out by one‐way ANOVA followed by Bonferroni's post hoc test

Subsequently, we further evaluated the suppressive effect of rutaecarpine on the Nox4‐ROS‐ADAM17 pathway in AAC‐induced hypertensive rats. O^2−^ and H_2_O_2_ are important components of ROS within the cardiovascular system. Considering that O^2−^ is short‐lived because it is rapidly transformed to H_2_O_2_ by superoxide dismutase in biological systems,^25^ the H_2_O_2_ content is used to evaluate ROS production in the left ventricle. As shown in Figure 5A, the H_2_O_2_ content was up‐regulated in the left ventricle of AAC group compared with that of the Sham group, whereas the H_2_O_2_ content was down‐regulated in the AAC rats treated with low or high dosage of rutaecarpine for 4 weeks. We next identified whether rutaecarpine decreased the Nox4 expression. Treatment with low or high dosage of rutaecarpine led to a decrease in the protein and mRNA levels of Nox4 in the left ventricle of the rats with AAC (Figure 5B and C). Concurrently, low or high dosage of rutaecarpine also diminished the ADAM 17 protein and mRNA levels in the left ventricle of the AAC rats (Figure 5D and E). For determining the ADAM17 activity, we further measured the TNF‐α protein level in the left ventricle using immunohistochemistry. As shown in Figure 5F, low or high dosage of rutaecarpine markedly reduced TNF‐α protein level in the left ventricle of AAC‐induced hypertensive rats, indicating that rutaecarpine decreased the ADAM17 activity in AAC‐induced hypertensive rats. And the result of TNF‐α mRNA is inconsistent with the inhibitory role of rutaecarpine in TNF‐ α protein level (Figure 5G). These results indicate that rutaecarpine inhibits the Nox4‐ROS‐ADAM17 pathway in the left ventricle of AAC‐induced hypertensive rats. Collectively, rutaecarpine exerts an inhibitory effect on the Nox4‐ROS‐ADAM17 pathway in hypertrophic cardiomyocytes.

### 3.5 Rutaecarpine blocked over‐activation of the ERK1/2 pathway

**Figure 6:**
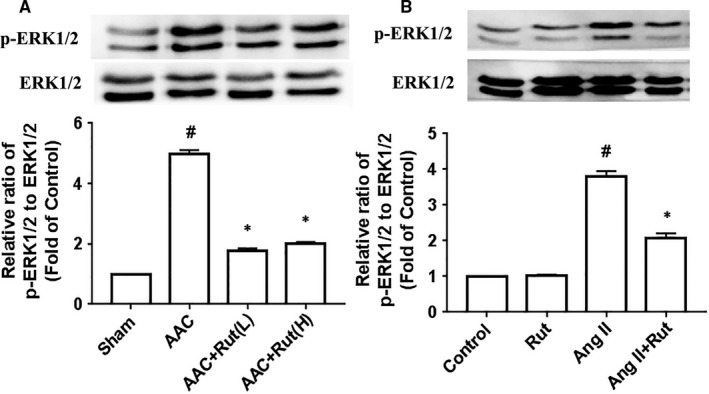
Rutaecarpine (Rut) blocked the over‐activation of the extracellular signal‐regulated kinase (ERK) 1/2 pathway in hypertrophic cardiomyocytes. A, ERK1/2 activity in the left ventricle of rats with abdominal artery constriction (AAC, n = 4 per group). Rut (L) represents low dosage of rutaecarpine; Rut (H) represents high dosage of rutaecarpine; ^#^ *P* < 0.05 vs Sham group, *P < 0.05 vs SHR group. B, ERK1/2 activity in primary cardiomyocytes treated with 100 nmol/L angiotensin II (Ang II) for 24 h (n = 3 independent experiments). After being pretreated with Rut (10 µmol/L) for 60 min, cardiomyocytes were then stimulated with 100 nmol/L Ang II for 24 h. ^#^ *P* < 0.05 vs Control group; *P < 0.05 vs Ang II group. The statistical analyses were executed using one‐way ANOVA followed by Bonferroni's post hoc test

We next determined whether rutaecarpine influenced the ERK1/2 pathway in hypertrophic cardiomyocytes because the ERK1/2 pathway has been proved to play a critical role in pathological cardiac hypertrophy.^26^, ^27^, ^28^ The left ventricle of the AAC rats displayed increased activity of ERK1/2 compared with the Sham group, whereas low or high dosage of rutaecarpine remarkably decreased the phosphorylation level of ERK1/2 in the left ventricle of AAC‐induced hypertensive rats (Figure 6A). Similarly, rutaecarpine down‐regulated the ERK1/2 activity in cardiomyocytes stimulated with 100 nmol/L Ang II for 24 hours (Figure 6B). Therefore, rutaecarpine suppresses over‐active ERK1/2 pathway in hypertrophic cardiomyocytes.

## 4 DISCUSSION

In this research, we discovered that rutaecarpine alleviated hypertensive cardiac hypertrophy in AAC‐induced hypertensive rats, that rutaecarpine suppressed Ang II‐induced cardiac hypertrophy and that rutaecarpine inhibited the Nox4‐ROS‐ADAM17 pathway and over‐active ERK1/2 pathway in hypertrophic cardiomyocytes.

NADPH oxidase 4 (Nox4) is a major enzyme that produce ROS within the heart.^29^, ^30^ Two contradictory viewpoints have been reported about the role of Nox4 in hypertensive cardiac hypertrophy. Sadoshima J's and Abboud HE's laboratories have stated that Nox4 advanced hypertensive cardiac hypertrophy,^30^, ^31^, ^32^ in contrast to the argument reported by Shah AM's team that Nox4 prevented hypertensive cardiac hypertrophy.^33^, ^34^ This discrepancy might be attributed to methodological differences among previous research, for example, different approaches to over‐express or disrupt Nox4 gene, different type and severity of hypertensive models (severe transverse aortic constriction vs abdominal aortic banding). Our previous studies have supported the positive role of Nox4 in hypertensive cardiac hypertrophy.^11^, ^17^ Additionally, Nox4 knockdown or over‐expression failed to cause significant change in blood pressure in hypertensive models.^32^, ^34^ Thus, Nox4 is a key mediator of hypertensive cardiac hypertrophy independent of blood pressure.

ADAM17 is a critical member of metalloproteinases family. This metalloproteinase mediates cell‐cell interactions, signaling and proteolysis of key cytokines, cytokine receptors and other targets.^35^, ^36^ ADAM17 advances hypertensive cardiac hypertrophy in spontaneously hypertensive rats and angiotensin II‐induced hypertensive mice^37^, ^38^; however, Fan D et al^39^ stated the contrary viewpoint that ADAM17 protected hypertensive cardiac hypertrophy at 5 weeks post‐transverse aortic constriction in mice. Furthermore, Shen et al^40^ reported another interesting outlook that lacking ADAM17 in vascular smooth cells improved hypertensive cardiac hypertrophy at 2 weeks post‐Ang II infusion in mice, but it failed to attenuate hypertensive cardiac hypertrophy on the 4th week of Ang II infusion. Several explanations for this disparity are possible. One reason may be that ADAM17 exerts a compensated effect on the heart via releasing the substrates in the distance, another possible explanation is the existence of different methods to disrupt ADAM17 gene. No significant change was observed in blood pressure in spontaneously hypertensive rats and vascular ADAM17 deficient mice infused with Ang II (1 μg/kg/min) for 2 weeks^37^, ^38^ although Shen et al^40^ found that lacking ADAM17 in vascular smooth cells only caused a transient reduction in blood pressure during the first week of Ang II infusion (1.5 mg/kg/day). These findings indicate that ADAM17 regulates hypertensive cardiac hypertrophy without changing blood pressure in the long term. Our previous findings demonstrated that Nox4 promotes Ang II‐induced cardiac hypertrophy by the ROS‐ADAM17 pathway in primary cardiomyocytes.^11^ Taken together, the Nox4‐ROS‐ADAM17 pathway is required for hypertensive cardiac hypertrophy independent of blood pressure.

The data in AAC‐induced hypertensive rats confirm the beneficial effect of rutaecarpine on hypertensive cardiac hypertrophy. The finding that rutaecarpine significantly inhibits hypertensive cardiac hypertrophy in AAC‐induced hypertensive rats supports its protective action against hypertensive cardiac hypertrophy. And this finding agrees with previous results that rutaecarpine attenuates isoprenaline‐ and AAC‐induced pathological cardiac hypertrophy.^9^, ^41^ In addition, we revealed that rutaecarpine inhibited the Nox4‐ROS‐ADAM17 pathway in the left ventricle of AAC‐induced hypertensive rats and primary cardiomyocytes stimulated with Ang II. Accordingly, blocking the Nox4‐ROS‐ADAM17 pathway is related to the protective action of rutaecarpine against hypertensive cardiac hypertrophy.

ERK1/2 is a key member of the family of mitogen‐activated protein kinase (MAPK). ERK1/2 activity within the heart is increased in aortic banding‐induced hypertensive rats and spontaneously hypertensive rats,^42^, ^43^, ^44^ in agreement with our findings that the heart exhibited elevated activity of ERK1/2 in AAC‐induced hypertensive rats. In vitro studies have verified the positive effect of ERK1/2 on cardiac hypertrophy in cardiomyocytes treated with endothelin‐1 or phenylephrine.^45^, ^46^ Nevertheless, ERK1/2 deletion in the heart failed to suppress hypertensive cardiac hypertrophy after 14 days of Ang II or phenylephrine infusion in mice.^47^ The possible explanation for this inconsistency might be that Ang II or phenylephrine infusion induces hypertensive cardiac hypertrophy through other signaling pathways such as p38 MAPK and JNK1/2 in mice with ERK1/2 deletion in the heart. However, ERK1/2 activation, induced by overexpression of MAPK1 within the heart, advances concentric cardiac hypertrophy.^26^, ^47^ Hence, ERK1/2 plays an important role in hypertensive cardiac hypertrophy. In the current study, we discovered that rutaecarpine markedly reduced ERK1/2 activation in the left ventricle of AAC rats and primary cardiomyocytes stimulated with Ang II. Overall, the inhibition of ERK1/2 pathway is associated the protective action of rutaecarpine against hypertensive cardiac hypertrophy.

In conclusion, we revealed that rutaecarpine attenuates hypertensive cardiac hypertrophy in AAC‐induced hypertensive rats; and inhibiting the Nox4‐ROS‐ADAM17 pathway and over‐activation of the ERK1/2 pathway might be involved in the beneficial role of rutaecarpine in hypertensive cardiac hypertrophy. These findings enhance our understanding of the mechanisms underlying the beneficial role of rutaecarpine in hypertensive cardiac hypertrophy, thus providing additional evidence for preventing hypertensive cardiac hypertrophy with rutaecarpine. However, the current study only examined the effect of rutaecarpine on the Nox4‐ROS‐ADAM17 pathway in hypertrophic cardiomyocytes. Further research should be undertaken to directly determine whether the pathway mediates the protective role of rutaecarpine in hypertensive cardiac hypertrophy in rats with constitutively active gene of Nox4 or ADAM17 in combination with rutaecarpine treatment.

## 5 DATA AVAILABILITY

All data used to support the findings of this study are available from the corresponding authors upon request.

## CONFLICTS OF INTEREST

The authors have no conflict of interest to disclose.
